# Editorial: Soft Tissue Biomechanics in Wound Healing and Prevention

**DOI:** 10.3389/fbioe.2022.897860

**Published:** 2022-04-05

**Authors:** Yih-Kuen Jan, Matthew J. Major, Fang Pu, Sharon Eve Sonenblum

**Affiliations:** ^1^ Rehabilitation Engineering Lab, Department of Kinesiology and Community Health, University of Illinois at Urbana-Champaign, Champaign, IL, United States; ^2^ Departments of Physical Medicine and Rehabilitation and Biomedical Engineering, Northwestern University, Chicago, IL, United States; ^3^ Jesse Brown VA Medical Center, Chicago, IL, United States; ^4^ Beijing Advanced Innovation Center for Biomedical Engineering, School of Biological Science and Medical Engineering, Beihang University, Beijing, China; ^5^ Rehabilitation Engineering and Applied Research Laboratory, The George W. Woodruff School of Mechanical Engineering, Georgia Institute of Technology, Atlanta, GA, United States

**Keywords:** blood vessel, constitutive properties, diabetic foot ulcers, pressure injury, pressure ulcers, skin breakdown, viscoelasticity, wound

Wound healing and prevention of chronic wounds are challenging issues in public health. Chronic wounds often result from pressure ulcers/injuries, diabetic foot ulcers, and skin breakdown of the residual limb in the case of limb deficiency. Pressure ulcers occur in people with limited mobility and impaired sensation, such as people with spinal cord injury, Alzheimer disease, and Parkinson disease (Sprigle et al., 2020). Diabetic foot ulcers are the result of complications caused by diabetes, including diabetic neuropathy and peripheral artery disease. Skin breakdown of the residual limb occurs due to repetitive loads transferred from the encapsulating prosthetic socket during load-bearing physical activities. Unfortunately, even after decades of efforts on the prevention and treatment of these wounds their incidence remains largely unchanged (Brienza et al., 2022).

Wounds are thought to result from prolonged, repetitive mechanical loads which may be either compressive or shear forces. The current clinical practice emphasizes the decrease of mechanical loads by providing support surfaces (wheelchair cushions, hospital mattresses, novel prosthetic socket materials and designs, and therapeutic insoles) to relieve points of high interface pressure between the device and the soft tissues of the individual. However, interface pressure alone does not fully describe the risk of wound development and thus preventive and treatment interventions based on this principle may not be fully effective (Jan and Brienza, 2006; Bader and Worsley, 2018; Gefen, 2019). In addition, current approaches often ignore the detailed biomechanical properties of compressed soft tissues consisting of the skin, subcutaneous tissue, fat, fascia, muscles, and blood vessels that are nonlinear, viscoelastic materials (Jan et al., 2013).

Computational and theoretical models can provide new insights on the development of wounds as well as optimization of the current clinical interventions. Rankin et al. used a high-velocity environmental debris in an animal cadaveric model to study mechanisms of traumatic amputation due to blast injury. The authors showed that high velocity sand blast is an independent mechanism of injury causing traumatic amputation. Kelly et al. explored the use of forefoot computational models from people with rheumatic arthritis (RA) to predict patient outcomes. Their magnetic resonance based models showed promise on predicting real life events in people with RA. Wang et al. performed a thermal analysis to predict blood flow of a foot using a vessel-porous media model and demonstrated exciting findings using thermal analysis to predict blood flow in 31 diabetic patients. These models allow for better understanding of the mechanisms of wound development and personalized prediction of outcomes.

Theoretically, a single mechanical overload can cause soft tissue damage, while repetitive sub-maximal mechanical loads can trigger adaptation (Liao et al., 2018). The adaptive response is known to be rate-dependent and is also affected by the magnitude of the mechanical loads and tissue viability of the individual. Various mechanical interventions, including exercise, have shown promise on improving wound healing and reducing risk for pressure injury. Roberts et al. showed a novel application of using low intensity vibration to improve angiogenesis and wound healing in diabetic mice. Ren et al. explored whether weight-bearing exercise increases risk for diabetic foot ulcers. The authors compared plantar microvascular function and tissue hardness in 80 participants with different volumes of weight bearing exercise. Their study demonstrated that higher volumes of exercise are associated with better microvascular function and lower plantar tissue hardness in people with type 2 diabetes. Duan et al. investigated the relationship between plantar tissue hardness and peak plantar pressure and pressure-time integral in people with and without Diabetic Peripheral Neuropathy. They provide evidence of the effect of soft tissue hardness on plantar pressure patterns.

Different patient populations and different sites of soft tissues can demonstrate very different structures, properties, and hence stress-strain relationships that respond differently to mechanical loads. Furthermore, the current understanding of soft tissue biomechanics is based primarily on the behavior of ligaments, tendons, and muscles under tension rather than on the mechanics of bulk soft tissue (consisting of skin, subcutaneous tissue, fat, fascia, muscle and blood vessels) under compression. Sonenblum et al. explored the relationship between adipose characteristics and pressure injury history in 43 wheelchair users. The adipose characteristics were obtained from MRI in a seated posture. The authors demonstrated that wheelchair users with a history of pressure injury had different subcutaneous fat characteristics than wheelchair users without a history of pressure injury. Similar to the findings of Duan et al., tissue properties, specifically intramuscular adipose, varied with years since injury or long term exposure to load. Qian et al. investigated the role of morphology and mechanical properties of plantar fascia in flexible flatfoot and plantar injury using B-mode and elastographic ultrasound. Another study conducted by Choi et al. used an optical coherence tomography-based air-jet indentation system to investigate the correlation between the indentation stiffness and type I collagen abundance, and organization of a wound in diabetic rats. Their study showed evidence of the relationship between indentation stiffness and collagen content of a diabetic wound.

Assessing microvascular networks embedded in the soft tissues can be used to evaluate risk for ischemic injury and wounds (Liao et al., 2013). Balasubramanian et al. reviewed skin blood flow in response to occlusion, pressure and temperature with the intention to establish the link between impaired skin perfusion and the development process of diabetic foot ulcers. Yeh et al. explored the use of cross-correlation and chaotic analyses of blood pressure and blood flow velocity to predict cardiovascular dysfunction and stroke. Their results show promise of using cardiovascular dynamics for early detection of cardiovascular related diseases.

It is evident from the articles in this research topic that biomechanical properties of soft tissues, including microvasculature embedded in soft tissues, affect the risk of developing pressure injury as well as wound healing. It is imperative to assess the changes of soft tissue in various pathological conditions as well as microvasculature in addition to traditional risk assessments, such as interface pressure. Mechanical stimulations (e.g., vibration and exercise induced mechanical stress to the weight-bearing tissue) show promise on promoting wound healing and decreasing plantar tissue stiffness associated with diabetes. More studies will be needed in order to translate these findings into clinical practice.
